# Secretome as a Tool to Treat Neurological Conditions: Are We Ready?

**DOI:** 10.3390/ijms242216544

**Published:** 2023-11-20

**Authors:** Andreia Valente da Silva, Inês Serrenho, Beatriz Araújo, Alexandre Martins Carvalho, Graça Baltazar

**Affiliations:** 1 Health Sciences Research Center (CICS-UBI), University of Beira Interior, 6201-506 Covilhã, Portugal; 2 Center for Neuroscience and Cell Biology (CNC-UC), University of Coimbra, 3004-504 Coimbra, Portugal; 3 Faculty of Health Sciences, University of Beira Interior, 6201-506 Covilhã, Portugal

**Keywords:** mesenchymal stem cells, secretome, conditioned medium, preconditioning, central nervous system

## Abstract

Due to their characteristics, mesenchymal stem cells (MSCs) are considered a potential therapy for brain tissue injury or degeneration. Nevertheless, despite the promising results observed, there has been a growing interest in the use of cell-free therapies in regenerative medicine, such as the use of stem cell secretome. This review provides an in-depth compilation of data regarding the secretome composition, protocols used for its preparation, as well as existing information on the impact of secretome administration on various brain conditions, pointing out gaps and highlighting relevant findings. Moreover, due to the ability of MSCs to respond differently depending on their microenvironment, preconditioning of MSCs has been used to modulate their composition and, consequently, their therapeutic potential. The different strategies used to modulate the MSC secretome were also reviewed. Although secretome administration was effective in improving functional impairments, regeneration, neuroprotection, and reducing inflammation in brain tissue, a high variability in secretome preparation and administration was identified, compromising the transposition of preclinical data to clinical studies. Indeed, there are no reports of the use of secretome in clinical trials. Despite the existing limitations and lack of clinical data, secretome administration is a potential tool for the treatment of various diseases that impact the CNS.

## 1. Introduction

Mesenchymal stem cells (MSCs) are valuable tools in regenerative medicine that show encouraging results [1]. In the target tissue, these cells respond to environmental signals that impose a regulatory action [2], which is related to the specific tissue pathology and leads to immune/inflammatory suppression [3]. Initially, it was thought that MSCs exerted their therapeutic effects through their migration to injured sites and their subsequent differentiation into target cells for tissue regeneration [4,5]. However, current studies point out that the beneficial effects of MSCs are attributed mainly to their paracrine activity [6] through the secretion of signaling molecules that induce regeneration of the host tissue [7].

The paradigm shift towards paracrine signaling as the main mechanism responsible for the therapeutic action of MSCs [7,8,9] has led to an increasing focus on the regenerative and immunomodulatory potential of the conditioned medium (CM) of these cells [10,11,12,13]. The CM represents the complete culture medium together with the secretome, i.e., all the molecules and biological factors secreted into the extracellular space by the cells. [5]. MSCs secrete a plethora of molecules crucial for biological functions [7] as diverse as cell growth, proliferation, differentiation, apoptosis, homeostasis, immune response, and angiogenesis [14,15,16,17,18,19,20,21]. Furthermore, the secretome is believed to be encoded by approximately 10% of the human genome and includes a range of serum proteins, growth factors, angiogenic factors, hormones, cytokines, extracellular matrix proteins, and even, in low abundance, lipid mediators, and genetic material [22], such as microRNAs (miRNA) [7]. These molecules are secreted by the MSCs into the medium through classical and non-classical secretion mechanisms [22], including protein translocation, exocytosis, and vesicle or exosome encapsulation [23].

The secretome tends to dynamically change its composition depending on the stimuli and the microenvironment to which the cells are exposed, i.e., depending on the environment or pathology they face, different pathways are activated to produce a particular molecular expression response [24,25]. Additionally, due to their high immunoplasticity, MSCs can change their phenotype (MSC1 or MSC2) in order to secrete pro-inflammatory or immunosuppressive mediators [26]. In addition, the MSCs and their secretome can modulate the immune system [27] and cellular phenotypes [28] of their target.

## 2. Secretome Composition

### 2.1. Soluble Factors

**Figure 1:**
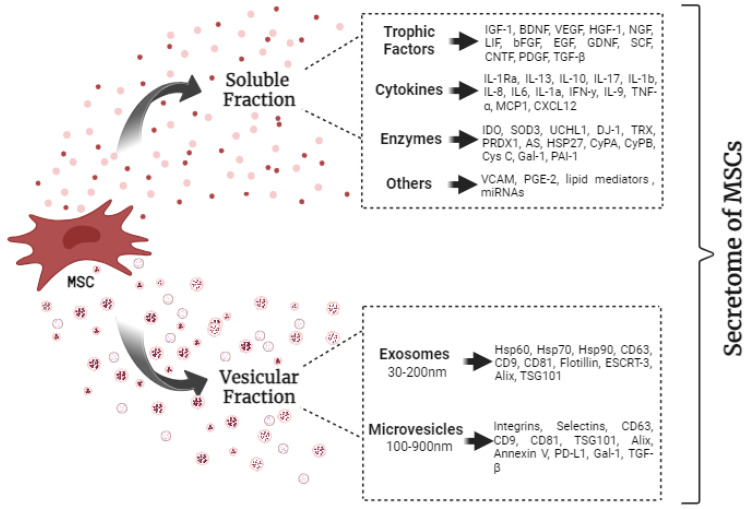
Summary of secretome composition and common surface markers of exosomes and microvesicles. IGF-1—insulin-like growth factor type 1; BDNF—brain-derived neurotrophic factor; VEGF—vascular endothelial growth factor; HGF—hepatocyte growth factor 1; NGF—nerve growth factor; LIF—leukemia inhibitory factor; bFGF—basic fibroblast growth factor; EGF—epidermal growth factor; GDNF—glia-derived growth factor; SCF—stem cell factor; CNTF—ciliary beurotrophic factor; PDGF—platelet-derived growth factor; TGF-β—tumor growth factor β; IL—interleukin; IFN-γ—Interferon gamma; TNF-α—tumor necrosis factor α; MCP1—monocyte chemoattractant protein 1; CXCL12—CXC motif chemokine ligand 12; IDO—Indoleamine 2,3-dioxugenase; SOD3—superoxide dismutase; UCHL1—ubiquitin carboxy-terminal hydrolase L1; DJ-1—Deglycase Protein; TRX—thioredoxin; PRDX1—pieroxyredoxin; AS—Albumine serine; HSP—heat shock protein; CyPA—Cyclophilon A; CyPB—Cyclophilon B; Cys C—cystatin C; Gal-1—galectin-1; PAI-1—plasminogen activator inhibitor-1; VCAM—vascular cell adhesion molecule; PGE-2—Prostaglandin E2; mRNAs—Messenger ribonucleic acid; CD—Protein-coding Gene; ESCRT—endosomal sorting complex required for transport; TSG101—tumor susceptibility protein 101; PD-L1—programmed death-ligand 1.

Many crucial biological molecules, such as cytokines, chemokines, enzymes, and trophic factors, as shown in Figure 1, are present in the soluble fraction of the secretome [9]. Although different populations of MSCs share phenotypic characteristics, their source [5,29], donor characteristics [30], preparation technique, culture conditions, and the number of cell passages [31] influence the composition of the secretome. Moreover, some of the secreted molecules are released only in certain microenvironments [32].

The cytokines responsible for immunomodulation described in the secretome include the interleukin 1 receptor antagonist (IL-1Ra) [11], the competitive inhibitor of interleukin (IL)-1, IL-13, IL-10, IL-17 [33], the CXC motif chemokine ligand 12, and the C-C motif chemokine ligand 2, also known as monocyte chemoattractant protein 1 [34]. After cleavage, mediated by metalloproteinases, this protein functions as an antagonist of chemotaxis and cell activation [32]. Pro-inflammatory cytokines such as IL1b, IL8 [11,12], IL6, IL-1a, interferon (IFN)-γ, and tumor necrosis factor α (TNFα) [33] and IL-9 [35] were also found in the secretome.

The presence of multiple trophic factors including insulin-like growth factor type 1 (IGF-1), brain-derived neurotrophic factor (BDNF) [36], vascular endothelial growth factor (VEGF) [37], hepatocyte growth factor 1 (HGF-1), nerve growth factor (NGF) [38], leukemia inhibitory factor [29], basic fibroblast growth factor (bFGF) [34], epidermal growth factor [29], and glia-derived neurotrophic factor (GDNF) [39] was identified in the secretome of MSCs, which is suggestive of its regenerative potential [32]. MSCs also secrete tumor growth factor B (TGF-B) [33], which has a key role in the activation of regulatory T cells, ciliary neurotrophic factor, and platelet-derived growth factor [33].

The secretome is also composed of enzymes. The cytosolic metabolic enzyme, indoleamine 2,3-dioxygenase [40], superoxide dismutase [17], and ubiquitin carboxy-terminal hydrolase L1, an enzyme mostly located in neurons and involved in the regulation of proteasomal degradation [13] were identified as components of the secretome.

A comparative study between the secretome of MSCs derived from three different sources, namely bone marrow (BM), adipose tissue (ADSCs), and umbilical cord perivascular cells, revealed differences in their composition. As an example, compared to the CM from BM-MSCs, ADSC-CM presents higher amounts of the angiogenic factors HGF and VEGF and of the adipokines plasminogen activator inhibitor-1 and IL-6. On the other hand, CM from BM-MSCs presents higher levels of stem cell factor, a chemokine involved in cell migration [41].

Despite the differences in the secretome composition, MSCs from all sources secrete important neuroregulatory molecules and anti-oxidative mediators, such as protein deglycase, thioredoxin, pieroxyredoxin, albumin serum, heat shock protein (HSP) 27, and anti-apoptotic molecules, such as Cyclophilin A, CyPB, cystatin C, or galectin-1 (Gal-1) [13]. Prostaglandin E2 [42] and the vascular cell adhesion molecule [21] are also secreted by MSCs. A summary of the secretome composition is shown in Figure 1.

### 2.2. Extracellular Vesicles

While for a long time, they were considered repositories of cellular “waste”, extracellular vesicles (EVs) derived from MSCs are now recognized as important players in intercellular communication [22]. Extracellular vesicles represent a sophisticated communication system between cells. Due to their ability to transport key molecules [32], including a variety of proteins, lipids, and functional RNAs (mRNAs and miRNAs) [22], EVs can affect the physiological and pathological mechanisms in the recipient cells [32]. This fraction is composed of membrane-coated vesicles enclosed by a lipid bilayer, rich in integrins, transpanins, and other ligands that support transport, adhesion, and endocrine effects, and contain bioactive components like lipids, proteins, or nucleic acids [43]. Moreover, EVs are classified according to their origin and size [9], i.e., with diameters ranging between 30 nm and 200 nm in exosomes [44] and 100 nm and 900 nm in microvesicles (MVs) [45].

#### 2.2.1. Exosomes

Exosomes, also known as nanovesicles [9], are formed intracellularly [32], have endosomal origin [43], and express as markers Hsp60 [46], Hsp70 [47], Hsp90 [46], CD63, CD9, CD81 [48,49], flotillin [50], the endosomal sorting complex required for transport 3 [51], Alix [52], and tumor susceptibility protein 101 (TSG101) [53]. Due to their small size and permeability to biological barriers [54], exosomes are studied as delivery systems for the treatment of tissues with restricted access to drugs, such as the CNS [55].

Exosome formation begins when a small amount of intracellular fluid is included in an early endosome [43], which, after undergoing a series of changes [32], becomes a late endosome and can then fuse with other endosomal membranes with consequent formation of intraluminal bodies, also known as multivesicular bodies. Then, the mature multivesicular bodies fuse with the plasma membrane, and the exosomes are released by exocytosis into the extracellular space [32,43].

Exosomes are released to the extracellular environment and participate in intercellular communication. The interaction between exosomes and target cells can occur by three different mechanisms: (1) direct fusion of exosomes with the cell membrane; (2) by endocytosis resulting in the delivery of the content into the intracellular medium of the target cell; and (3) interaction between lipid-binding receptors present in the cells and the lipids of the exosomal membrane [56].

Due to their lower propensity to trigger innate and adaptive immune responses and inability to directly form tumors, exosomes can be considered a safer strategy than cells [57]. Additionally, these structures can store and transport molecules without losing their function, thus conserving their cytoprotective properties and benefits with the activation of pathways where needed [58]. Through exosome encapsulation, therapeutically relevant molecules (proteins and nucleic acids) are protected from degradation. Moreover, exosomes exhibit a transport system that allows the delivery of small proteins and different RNAs (mRNA, tRNA, miRNA, and other non-coding RNAs) involved in the MSCs’ immunoregulatory response [59]. From a preparation point of view, exosomes are stable and can be stored in much smaller volumes than CM [5]. However, exosome-based therapies may also carry hazards, such as the unregulated transmission of genetic information between cell populations [60].

#### 2.2.2. Microvesicles

Microvesicles, also known as ectosomes [9], are more heterogeneous than exosomes [32] and represent medium-sized EVs. These vesicles have a range of sizes that vary from 100 to 900 nm [45], with the most frequently mentioned size being between 200 and 400 nm [61,62,63]. These EVs originate from the plasma membrane of the cell, and their common surface markers are specific integrins (e.g., macrophage-1 antigen), selectins (p-selectin) [9,32], CD63 [64], CD9, CD81, TSG101, Alix [45], and Annexin V [65]. These vesicles also express some regulatory molecules, such as the programmed death-ligand 1, Gal-1, and TGF-β [66].

The process of MVs formation requires the reorganization of the cytoskeleton, i.e., phosphatidylserine is moved towards the outer layer to induce localized curvature, and consequently, cytosolic proteases, such as calpain and gelsolin, are activated in order to disrupt the protein network of the actin cytoskeleton, allowing membrane division [32]. Their release is dependent on intracellular calcium, and their membrane is usually enriched in lipids such as cholesterol, sphingomyelin, and ceramide [67]. MVs transport secondary metabolic products, lipids, proteins, and nucleic acids like mRNA, miRNA, and other non-coding RNAs [9].

#### 2.2.3. miRNAs

A lot of focus has recently been placed on the nucleic acid composition of EV and the involvement of these structures in RNA and miRNAs transport and release to recipient cells [32] and also in their role in the specificities of secretomes [7].

It is worth noticing that EVs are particularly rich in 3′untranslated region (3′UTR) mRNA fragments with multiple miRNA binding sites. The RNA fragments compete with cellular RNA to bind to miRNAs or proteins contained in the recipient cell and consequently regulate cellular stability and protein expression [32]. However, these RNA fragments play a marginal role compared to the post-transcriptional inhibition of gene expression carried by miRNAs [32].

There is a repertoire of miRNAs selectively transported by EVs [68], which, due to their incorporation in vesicles, can circulate in the blood without being degraded by RNases [32]. Initially, it was suggested that neither tissue-specific nor donor-specific microenvironment traits significantly affect the expression of miRNA levels when compared to the other components of the secretome. However, when comparing EV preparations from different tissues, significant differences were found [68,69]. EVs include miRNA with angiogenic, immunoregulatory, and regenerative properties, such as miR-23a -3p, miR-199a, and miR-130a-3p [70].

## 3. Advantages of the Therapeutic Use of the Secretome Compared to MSCs

Even though MSCs show great therapeutic potential, the outcomes are often inconsistent, and clinical and preclinical studies sometimes report low effects [55]. Despite the intense study in the field and its important advances, there are still obstacles to their clinical use [43]. The use of MSCs has potential drawbacks, including host rejection, ectopic tissue formation [9], detrimental effects on the pulmonary microvasculature, which may lead to their entrapment in these tissues [71], and pro-tumoral activities [72]. Other limitations are the increased risks of arrhythmia [73], ossification, and calcification [74]. Moreover, some cryopreservation protocols may reduce the viability and functions of MSCs [75].

While the intravenous administration of human MSCs is typically regarded as safe, with only minor adverse effects such as fever and post-infusion site reactions, there is still a risk of more severe adverse effects, such as thrombosis or adverse inflammatory outcomes [43].

These are some of the obstacles that contributed to the limited clinical application of MSCs to date [9], and, as a result, there has been a growing interest in the use of cell-free therapies in regenerative medicine [22]. The use of the secretome offers important advantages over stem cell-based applications [5] and can even overcome some of the important limitations mentioned before. Being a cell-free strategy, the secretome circumvents the potential adverse effects of cell transplantation [43].

While the administered MSCs produce an unknown pool of bioactive factors [43] influenced by the microenvironment, the MSC secretome composition can be evaluated for safety, dosage, and potency in an analogous manner to conventional pharmaceutical agents [5,43]. This also contributes to the standardization of the protocol, which can increase the scalability and reproducibility of the results [9,43]. Additionally, CM-MSCs can be stored for long periods without losing their potency, without the addition of potentially toxic cryopreserving agents for long periods [8].

The secretome provides a convenient source of bioactive factors [5], and the biological product obtained for therapeutic applications can be modified for specific effects [76].

MSCs require expansion in culture until they reach the optimal number of cells for transplantation [77], and although they can be prepared in advance, the secretome is a more practical strategy since it is immediately available when needed for the treatment of acute conditions [22], such as cerebral ischemia, myocardial infarction, and spinal cord injuries [5,8].

## 4. The Therapeutic Use of the Secretome in Neurological Diseases

Brain tissue is formed by complex and integrated relationships between different cell types. Neurons, for example, are responsible for neurotransmission, while glia [54] plays an important role in supporting neuronal activity and mediating neuroinflammation [78]. Tissue injury triggers events that promote repair and regeneration while increasing inflammation in the tissue. Some of the damage subsequent to CNS insult involves disruption of the blood–brain barrier (BBB), inflammation, edema, ischemia, excitotoxicity, increased levels of free radicals, and altered cell signaling and gene expression [79]. These events, consequently, can lead to cell death, which, in turn, may lead to both physical and cognitive sequels [80]. The development of therapeutic approaches that limit brain damage and promote its recovery is therefore crucial.

Recently, through clinical and pre-clinical studies, the secretome and its products have been studied as a possible therapeutic strategy for neurological diseases. This section will discuss the main results obtained with these approaches in clinical studies and in vivo models of disease.

### 4.1. Clinical Studies

Being a recent strategy, the number of completed clinical studies evaluating the potential of secretome administration in CNS diseases is limited. So far, the only clinical trial conducted on neurological diseases was performed by Dahbour, et al. (2017) in patients with multiple sclerosis (NCT01895439) [81]. In this study, the applicability, safety, and efficacy of the administration of autologous BM-MSCs in combination with their CM was evaluated, being later administered intrathecally one month after BM-MSCs administration. The researchers reported that there was a correlation between decreased brain lesions and elevated levels of factors such as IL-6, IL-8, and VEGF in the CM of the administered cells. Despite some minor adverse effects, the protocol used was safe, feasible, and possibly effective in stabilizing the disease and reversing its symptoms.

Three other studies using the CM from MSCs are in the recruitment phase for the treatment of acute ischemic stroke (NCT05008588) [82], Multiple Systemic Atrophy (NCT04876326) [83], and Cerebral Palsy (NCT04314687) [84]. However, none of the studies uses CM alone but rather in combination with the administration of MSCs. This fact may be related to some of the limitations found in the use of the secretome, which will be covered in a subsequent section.

As mentioned earlier, secretome products, such as EVs, have also been studied. No completed clinical studies for neurological diseases were found with the use of EVs. However, three studies are in the recruitment phase for the treatment of IS (NCT03384433) [85], Alzheimer’s disease (AD) (NCT04388982) [86], and the study of neuroprotective effects in premature newborns (NCT05490173) [87]. In contrast to what was found for the secretome, all these studies propose the use of MSCs-derived exosomes alone, without combining it with an associated strategy.

### 4.2. Preclinical Studies

#### 4.2.1. Alzheimer’s Disease

Alzheimer’s disease (AD) is a form of dementia that involves the extracellular deposition of senile plaques enriched in β-amyloid aggregates and intracellular tau-enriched neurofibrillary tangles, affecting synaptic function [88]. The therapeutic use of the secretome has been studied in preclinical models of AD. Several authors observed that the use of the secretome switched the environment of the brain tissue from pro-inflammatory to anti-inflammatory [89] by decreasing the number of reactive glial cells [88]. Moreover, this strategy restored neuronal structure [39] with an increase in neuronal density both in the cortex and the hippocampus [88]. Recovery of both cognitive and motor deficits was also observed after secretome administration [39,88,89]. Additionally, Mita et al. (2015) reported an increase in neurotrophic factors and neuroprotective effects against glutamate neurotoxicity [89], and Santamaria et al. (2021) reported a decrease in β-amyloid oligomers (Aβ) [88]. Moreover, Venugopal et al. (2022) studied the effects of CM applied to animal models suffering from hippocampal neurodegeneration, reporting that this strategy reduced neuroinflammation, promoted hippocampal neurogenesis, and induced anti-apoptotic effects [90].

When comparing the data on the effects of EVs with the data from the use of the whole secretome, despite the concordance with some of the results previously mentioned, it was possible to observe the impact of EV administration in reducing the accumulation of β-amyloid aggregates, either by promoting the secretion of enzymes that degrades Aβ or by activating the SphK/S1P signaling pathway [91,92,93]. Liu et al. (2022) also observed a decrease in hyperphosphorylated *tau* in the hippocampus and an increase in the expression of BDNF, an important regulator in neuronal plasticity [92]. Along with the previously mentioned outcomes, Niu et al. (2020) reported increased adiponectin production [94], a protein involved in the homeostasis of metabolic processes that has been correlated both with stress and the development of psychiatric diseases [95] and in the regulation of neurogenesis [94].

#### 4.2.2. Multiple Sclerosis

Multiple sclerosis, a disease caused by progressive demyelination, is one of the most prevalent autoimmune illnesses. This disease presents as hallmarks of increased oxidative stress, ion and mitochondrial channel dysfunction, and excessive excitatory neurotransmission [52].

Borhani-Haghighi et al. (2020) and Zhang et al. (2022) evaluated the potential of the secretome or EVs in animal models of Experimental Autoimmune Encephalomyelitis (EAE). Both studies showed that animals treated with EVs present increased remyelination, recovery of neurological functions, reduction of functional deficits, and reduction of inflammation and gliosis [52,96]. Moreover, Zhang et al. (2022) observed an increased polarization to the M2 phenotype of microglia, with a consequent increase in anti-inflammatory cytokines such as IL-10 and TGF-β [52].

#### 4.2.3. Parkinson’s Disease

Tremors and bradykinesia are hallmarks of Parkinson’s disease, a neurodegenerative condition whose clinical features include the gradual degradation of the nigrostriatal pathway, with consequent striatal dopamine depletion [97]. Chen et al. (2020) observed that the administration of exosomes to animal models of PD decreased the number of apoptotic dopaminergic neurons while increasing dopamine levels and improving behavioral outcomes [98]. Xue et al. (2021), on the other hand, reported increased expression of ICAM1 in the corpus striatum and substantia nigra and enhanced expression of CD31 in the corpus striatum area and promotion of angiogenesis [97].

#### 4.2.4. Stroke

A stroke is caused by a blockage or rupture of a blood vessel in the brain, which leads to oxygen and nutrient deprivation in a certain area of the brain [99]. Due to the limitations of current therapies, it is also one of the leading causes of long-term disability [100].

Most of the studies reported functional recovery after the administration of CM in animal models of stroke [100,101,102,103,104]. Additionally, an increase in neurogenesis [102,103], angiogenesis and its markers [100,104], attenuation of microglia activation and macrophage infiltration [102], and decreased expression of apoptotic proteins [103] were reported. Regarding the impact of CM administration on the ischemic lesion volume, some studies reported a decrease in lesion volume after treatment with CM [101,103,104], while others observed no effects of CM administration [100,102] in this parameter.

Regarding the use of EVs as a potential therapy for stroke, most studies reported decreased lesion volume [105,106,107,108,109], reduction of weight loss [110], improved cognitive and motor function [105,106,107,108,111], induction of neuroprotection [111], and promotion of angiogenesis and neurogenesis [106,110,111]. Modulations of immune responses after EV administration [111], namely decreased levels of IL-1β, TNF-α [106], decreased accumulation of Iba1^+^ cells [110] and proteins associated with the inflammasome and pyroptosis, and polarization of microglia towards the M2 phenotype, were also reported [107].

#### 4.2.5. Hypoxic-Ischemic Encephalopathy

Neonatal Hypoxic-Ischemic Encephalopathy (HIE) is one of the most serious complications in the perinatal period. During an HI event, the amount of oxygen and glucose reaching the brain is insufficient to meet the metabolic demands, leading to a series of biochemical reactions that cause widespread brain damage and consequent cognitive and motor deficits [112].

Wei et al. (2009) and Huang et al. (2022) analyzed the impact of administering the secretome of MSCs in an animal model of HIE, identifying an attenuation of brain damage and an improvement in cognitive and motor deficits [36,112]. Additionally, protection against glutamate-induced excitotoxicity [36] and a decrease in the number of cells positive for caspase-3, GFAP, vimentin, and Iba-1 were reported [112].

At the structural level, administration of EVs or exosomes reduced the volume of brain damage of both white [113] and gray matter [114], hypomyelination [113,115], and brain edema [116]. The use of exosomes alone was shown to impact neuroinflammation by reducing the secretion of the inflammatory factor IL-6 [116,117], IL-1β, and TNFα [116] and increasing TGF-β [117]. Moreover, a decrease in Iba1^+^/CD68^+^ cells was also reported [117]. Although Ophelders et al. (2016) observed prevention of cortical dysfunction determined by evaluation of seizure burden, there was no protection against apoptosis or neuroinflammation [113]. Exosomes were also shown to improve functional recovery [114], namely cognitive deficits [117].

#### 4.2.6. Traumatic Brain Injury

Traumatic brain injury (TBI) can lead to the occurrence of acute (days to weeks) or chronic (months to years) symptoms [118] associated with neurological, cognitive, and motor deficits. Although advances were made, an efficient therapeutic approach prompting the functional recovery of these individuals has not yet been achieved [118]. Preclinical data concerning the use of secretome as a potential therapy for this injury is scarce. Nevertheless, it was reported that the administration of the secretome in TBI models decreased the number of apoptotic cells; attenuated cerebral infarction volume; increased neurogenesis [37,119]; up-regulated proteins involved in this process, such as phosphorylated p-β-catenin, Pox1, Neurog2, and NeuroD1 [25]; enhanced neural stem cells differentiation in the dentate gyrus; and improved the migration and maturation of new neurons [25]. Administration of secretome was also shown to reduce the expression of GFAP immunoreactivity [120], one of the prominent serum cerebral biomarkers following TBI [121]. Moreover, improvements in the motor and cognitive functions were observed [25,37,119,120].

Administration of EVs to TBI models had a positive impact on the pathological pathways that lead to tissue destruction and inflammation and on learning functions [122]. Several encouraging outcomes were found in studies assessing exosomes as a therapeutic strategy for TBI. Improvements in cognitive [44,123,124] and motor impairments [44,48,123,124,125,126] along with increased angiogenesis [123,124] and neurogenesis [44,123,124,126] and decreased cell apoptosis [44,47,124,125,126,127,128] were observed. Furthermore, decreased brain inflammation [123,124,125,126,127] and reduction in pro-inflammatory factors such as TNF-α, IL-1β, and IL-6 [47,127] through the inhibition of the NF-κB signaling pathway [47] were reported. Additionally, a reduction in microglial activation [125] with decreased CD68-positive cells [48], reduction of glutamate-induced excitotoxicity [127], and iNOS expression, as well as increased expression of Arg1, STAT3, and miR-181b levels, were observed [47], being the last one suggested as a crucial element in the regulation of microglia phenotype and neuroinflammation. In contrast to Zhuang et al. (2022) and Ni et al. (2019), who observed a decrease in lesion size [125,127], Zhang et al. (2015) reported no appreciable differences [123].

Williams et al. (2020) examined the impact of exosome administration in pigs after inducing TBI followed by hemorrhagic stroke. The authors reported that administration of exosomes decreased intracranial pressure; increased cerebral blood flow; reduced swelling, brain damage, and the number of apoptotic cells; and restored BBB. These changes were associated with an increase in neurological recovery [121,129,130]. Moreover, it was also possible to verify decreased levels of IL-1, IL-6, IL-8, IL-18, GFAP [121], lipocalin 2, HIF1A, TNF, proline, and glutamate [130] and increased expression of genes related to neuronal development, neurogenesis, cell cycle progression, microtubule dynamics, neuronal differentiation, and nervous system development [121,130].

#### 4.2.7. Other Pathologies

**Figure 2:**
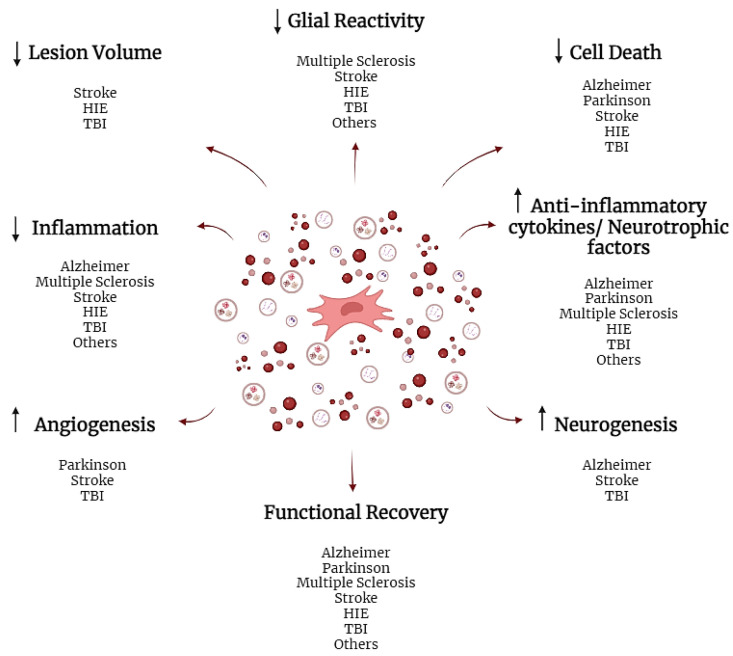
Summary of the main outcomes observed with the administration of secretome or its vesicular fraction for the treatment of neurologic disorders. The arrows indicate an increase (↑) or decrease (↓) in the respective processes or molecules.

The secretome and its components have also been used in preclinical studies of other conditions that affect the nervous system. Within these are included animal models that present impaired astrocytic exocytosis and function [131], spinal cord injury [132], neuropathic pain [133], and peripheral nerve injury [46]. The main findings reported included weight gain [133], recovery of motor function [46,132], reduction of allodynia [133], reduction in inflammation/overexpression of inflammatory cytokines [46,132], glial activation [46], promotion of GAP-43-labeled axonal fiber growth [132], increased expression of IL-10 and BDNF [46], and promotion of subgranular zone proliferation [131]. A resume of the main outcomes observed with the administration of secretome or its vesicular fraction is shown in Figure 2.

## 5. Preconditioning

As previously mentioned, although MSCs have an innate potential to induce and/or contribute to regeneration, it is now known that this potential is significantly influenced by factors such as the tissue source of the MSCs, the age and general health of the MSC donor, the cell culture conditions such as the culture medium, the number of cell passages [29,30,31,134], oxygen levels, or the presence of a pro-inflammatory environment [76].

Despite paracrine signaling pathways being one of the main mechanisms of action of MSCs when transplanted, only a few groups have studied how preconditioning of MSCs affects their secretory profile [76].

Several strategies have already been studied to improve MSCs’ lifespan or to modify their secretome in order to achieve a greater therapeutic potential [22] by enhancing their immunomodulatory characteristics, migratory capability, and/or hypoimmunogenicity [55]. Thus, to perform preconditioning of MSCs, various methods have been investigated, including (a) 3D culture [135,136] and/or its administration on 3D scaffolds [118,137]; (b) exposure to pharmacological or chemical compounds [138,139]; (c) exposure to cytokines, chemokines, or growth factors [140,141,142]; and (d) culture under hypoxic conditions [37,143,144].

### 5.1. Three-Dimensional Culture and Three-Dimensional Scaffolds

The three-dimensional culture of MSCs has been studied as a possible preconditioning alternative to the conventional culture approach. This procedure involves matrix-free 3D cell culture, such as spheroids, or the use of different scaffolds made of synthetic or natural materials [137] to improve its effectiveness after administration [145].

Administration of spheroids of MSCs in animal models of pathologies affecting the brain tissue increased both the proliferation of cells after transplantation and the expression of angiogenic factors, like VEGF [146]. In addition, the reduction of brain infarct volume, increase in neuronal differentiation capacity, and increase in expression of anti-inflammatory and growth factors, such as IL-10, IL-11, IL-13, NT3, bFGF, and GDNF, were observed [146].

Regarding the administration of CM, Chen, C. et al. (2022), and Liu, X., et al. (2022), resorted to the use of 3D scaffolds carrying the secretome of MSCs. Despite the different biomaterials, animal models, and protocols used in the two studies, they obtained identical outcomes. The injury size decreased, and nerve fiber regeneration and remyelination were promoted [118,137]. An increase in neuronal differentiation and angiogenesis and a decrease in the levels of microglial activation, cell death, and pro-inflammatory factor production were reported [118]. Additionally, improvement in locomotor function and physiological activity [137] were also observed.

Still, within this strategy, to understand what effects underly the use of EVs isolated from CM obtained from the 3D culture of MSCs (free of 3D scaffolds), Cone, A.S., et al. (2021), showed an improvement in phenotypic behaviors resulting from AD, reduction of Aβ plaque deposition, GFAP levels, and its co-localization with thioflavin S, suggesting a decrease in inflammation [136]. In this sense, Zhang, Y., et al. (2017), isolated exosomes from MSCs cultivated on 3D scaffolds and obtained outcomes comparable to those already discussed. The authors reported recovery of cognitive and motor functions, as well as increased angiogenesis, neurogenesis, and a decrease in inflammation. Contrary to the outcomes obtained after administration of the cells or CM with the same strategy, the authors did not observe changes in the volume of the cerebral infarct [49] with the use of EVs.

### 5.2. Pharmacological or Chemical Compounds

Preconditioning with pharmacological or chemical compounds is one of the methods being investigated to improve the efficacy of treatment with MSCs, the secretome, or its components. The compounds tested include valproate, lithium chloride [139,147], hydrogen sulfate [148], tetramethylpyrazine [149], calpain inhibitors [150], thrombin [151,152], fasudil [153], rapamycin [154], roxadustat [155], cobalt chloride [138], and salidroside [156]. Despite their differences, all have been proposed to trigger anti-inflammatory [150,156], neuroprotective [138,148,150,151,152,156], and other regulatory effects, which influence both the activation of important signaling pathways, cell migratory mechanisms and differentiation, ultimately leading to the regeneration of injured tissue [139,147,148,149,153,154,155].

Despite the different protocols, preconditioning techniques, cell sources, and therapeutic applications, it was possible to observe some consistency in the outcomes when the preconditioned MSCs were administered. Most of the studies reported increased functional recovery at the behavioral level [139,147,148,149,150,151,153,155] and decreased brain infarct volume [139,148,150,151], degeneration [147], and cell death [148,150,151,155]. Additionally, reduced neuropathological signs, either by decreasing the secretion of pro-inflammatory factors (IL-1α [151,153], IL-1β, IL-6, and TNF-α [150,151,153,155]), microglial activation [150,151,155], and improved cellular morphology [148], were also mentioned in these studies. Increased secretion of anti-inflammatory cytokines [150,154], growth factors [148,153], and angiogenesis [139,148,149], expression of proteins such as SDF-1 and CXCR4 [149], and improved homing efficiency [139,149] and cell survival following transplantation [147,149,152] were also shown.

Similar outcomes were obtained with the secretome from preconditioned cells. Improvements in brain damage and cognitive deficits [138], as well as lower levels of apoptosis, microglial reactivity, and neuroinflammation [156], were reported. Along with these findings, Day, Y. et al. (2017), showed an increase in the expression of the protein GluR2 [138], which is crucial for the survival of neurons [157].

### 5.3. Cytokines and Growth Factors

Preconditioning with cytokines and growth factors is another method that aims to increase the effectiveness of MSC transplantation and the release of bioactive molecules crucial for mitigating brain injuries. This method includes the use of SDF-1α [158], IGF-1 [140], TNF-α alone [159], or in combination with IFN-γ [141,160,161,162], IL-1α [142], and BDNF [163,164].

Preconditioning MSCs using this method enhanced functional and cognitive recovery [158], increased homing [140], neurogenesis [143,162], and angiogenesis [158], as well as decreased microglial activation [140]. Boroujeni, F.B. et al. (2020), also identified a recovery of axonal structure and reduction of myelin sheath damage. Regarding the application of preconditioned CM, functional recovery, both at the motor [142,162] and cognitive [162] levels, mitigation of visual deficits [165], and reduction of brain infarct volume [142] were reported. Jha, K.A, et al. (2021), also showed the attenuation of aquaporin 4(AQP4) levels [159], a protein that has been linked to the development of edema in pathological circumstances [166]. Reduction of cell death [162], decreased neuroinflammation [162], and the transcription of genes involved in its onset [165], oxidative stress [159,161,162], and microglial activation [160,161,165] were consistently reported. Moreover, administration of preconditioned CM increased the neuroprotection [142,164].

The administration of EVs, namely exosomes, isolated from preconditioned cells using the same strategy, led to neuronal regeneration [141,163], decreased cell death, and promoted functional recovery [163]. The decline in microglial activation and decline in the secretion of pro-inflammatory cytokines, as well as an increase in anti-inflammatory cytokines [141] and expression levels of miR-216 a-5p [163], a miRNA that is involved in the reduction of inflammation [167], were also reported.

### 5.4. Hypoxia

One of the most studied methods of enhancing the therapeutic potential of MSCs and their secretome or its components has been hypoxia. The term hypoxia, when employed in the context of cell culture, is routinely used to refer to exposure to oxygen pressure levels between 0 and 10%, compared to the normoxic pressure of 21% [76]. Numerous authors showed the benefits arising from the treatment with MSCs preconditioned with hypoxia [168], which included improved neurogenesis [144,169,170,171], differentiation [144], and even a subsequent improvement in transplanted cells’ survival [172] and their homing [170,172,173]. A decrease in cell death [168,169,171,173,174], brain infarct volume [170,172,173], microglial activation [168,174], and various factors that lead to tissue inflammation [144], such as TNF-α [168,172,174], IL-6, IL-1a [174], IL-1b [168,174], and S100B [172], a cytosolic calcium-binding protein family member [175] that has been described as being involved in inflammatory processes [176], were all observed. Several studies also reported a decrease in neurological deficits [169,173,174], increased angiogenesis [144,170,171] with consequent secretion of Ang-1 [144], and regenerative growth factors such as GDNF, BDNF [144,170], and HIF-1α [144,171], as well as improvements in functional recovery [144,170,174].

When CM was harvested from cells preconditioned with hypoxia and administered in animal models of diseases affecting brain tissue, the outcomes observed were consistent with those seen when the cells were administered. Chang, C.P. et al. (2013), Jiang, R.H., et al. (2019), and Xu, C. et al. (2020), reported an attenuation of the infarct volume and decreased cell death [119,177,178]. The authors also described, in addition to a reduction in pro-inflammatory factor secretion and microglial activation [178], the recovery of both motor and cognitive functions [119,177] and an increase in neurogenesis [119] and angiogenesis [177].

Data regarding the administration of exosomes isolated from hypoxia-preconditioned MSCs also demonstrate the positive effects of this approach in different models of disease. Cui, G.H. et al. (2018), Nalamolu, K.R, et al. (2019), as well as Liu, X. et al. (2022), with the latter group using larger animal models (Beagles) were able to observe a functional recovery with this type of therapy [143,179,180]. A decrease in pro-inflammatory factors has also been identified [146,181], with Cui, G.H et al. (2018), reporting an inhibition of NF-κB pathway activation and microglial activation and an increase in the secretion of anti-inflammatory factors [143]. Liu, X., et al. (2022), identified an increase in regeneration, myelination, neurogenesis, angiogenesis, and attenuation of both the volume of brain infarction and cell death [179].

### 5.5. Other Methods

Although the preconditioning techniques already covered in this section are the most widely employed, there are other types of preconditioning being studied. Among these is the incubation with tissue extracts from lesioned brains [25,182,183] and the use of a low-intensity pulsed ultrasound [184]. Due to its capacity to cause therapeutic effects without causing a biologically meaningful rise in temperature, the use of low-intensity ultrasound has attracted interest [181]. Ning, G.Z, et al. (2019), reported that by administering cells stimulated with low-intensity pulsed ultrasound, rodents presented a higher functional recovery after SCI compared to those that were administered with cells not subjected to stimulation. The authors also observed a reduction in lesion size, activation of the microglia, and increased expression of BDNF, NGF, and cells positive for neurofilament 200 [184], whose expression has been linked to the number of mechanoreceptors with myelinated fibers [185].

As previously mentioned, MSCs respond to stimuli produced by their microenvironment. This response can be used to enhance their efficiency in transplantation or induce a different secretory profile. Zheng, J., et al. (2022), pretreated MSCs using rat cerebral infarction tissue to simulate the complex microenvironment in the injured brain. The author’s findings were encouraging, showing elevated expression levels of factors such as BDNF, bFGF, HGF, VEGF, and CXCR4, decreased brain infarct volume and cell death, with consequent attenuation of neurological damage and cell proliferation [182]. With the same aim, Liu, X. Y., et al. (2020), administered CM from MSCs preconditioned with traumatically injured brain tissue extracts. This approach led to a reduction in cognitive deficits; increased excitatory postsynaptic potential slope; and increased proliferation, differentiation, and subsequent migration of new neurons [25]. Ye, Y. C., et al. (2022), reported a reduction in lesion volume and cell death after administering exosomes isolated from cells stimulated with cerebral infarct tissue. The authors also identified improvements in neurological function and cerebrovascular remodeling [183]. This type of strategy will help identify which signaling molecules released by the injured tissue influence the MSCs and lead to an increase in their therapeutic effect. A summary of the main outcomes observed by the different preconditioning methods is presented in Table 1.

## 6. Preparation of the Secretome

Despite the findings from studies assessing the impact of the secretome administration being consistent and showing positive outcomes in many neurological diseases, the secretome composition can differ. Indeed, there is a significant heterogeneity in the protocols of secretome preparation, including the number of cells used in the culture, the culture media used, the method used to recover the supernatant (i.e., CM), and even the method used to concentrate the CM. This point is crucial when the objective is to test its therapeutic efficacy and influence the volume of CM administered, the number of administrations, and its therapeutic effects. This section addresses the main differences and similarities found in the methodology of secretome preparation.

### Secretome Formulation

Although the basis for secretome production throughout all studies involves cell culture, CM production and/or extraction, concentration (i.e., centrifugation or ultrafiltration), and storage, one of the major variations is the number of cultured cells used. This step is vital for the composition of the secretome, namely the amount of protein present in the CM. The lack of consensus on this aspect leads to cell cultures with densities varying from 2.5 × 10^3^ to 2 × 10^10^ cells, as shown in Table 2. In some studies, the number of cells and the total amount of protein in the CM were used as a control for the amount of CM prepared, as is the case of Farfán, N., et al. (2020). In this study, the authors indicate that the volume administered, in this case, 16 µL, corresponded to approximately 6 µg of protein produced by 2 × 10^5^ cells [162]. For most of the studies, it is impossible to infer the number of cells responsible for the conditioning of the medium. Besides the initial number of cells, the time in culture and the number of passages are equally important for the composition of the CM.

The composition of the culture medium is also a key factor for the growth of MSCs without the loss of their features [186] and for reproducibility in studies [187]. There are a variety of options that might be used, either for the culture of MSCs or for the replacement of the medium after reaching the appropriate confluence for CM collection. When performing this step, a variety of media, such as Eagle’s basal medium (BME) [36,120], Dulbecco’s modified Eagle’s basal medium (DMEM) [104,132], DMEM/F12 [102], alpha minimal essential medium (α-MEM) [16,103,120], and neurobasal-A medium [16,39,131] are commonly used. All of these media were supplemented with serum (usually bovine or human), which is added in order to provide nutrients for cell growth, adhesion, and proliferation [188]. The use of serum brings disadvantages such as a higher risk of contamination, the presence of growth inhibitors, and lack of uniformity in composition [189], which in turn causes phenotypic and population kinetic diversity in the MSCs produced and in their secretome [190], and even the potential interference with the purification and isolation of CM products [189]. Serum-free media have also been employed to improve safety, efficacy, consistency, and reproducibility [190].

The term “serum-free media” refers to a medium chemically defined that does not require serum supplementation, although it may contain small protein fractions (such as animal tissue or plant extracts) in its composition [187]. The use of this type of medium is rising, especially when the study aims to collect CM for later use. Many authors used both types of media, as in the cases of Faezi, M et al. (2018), and Liu, X.Y.E., et al. (2020), they start their cultures in a serum-supplemented medium before switching to a serum-free medium once the desired cell confluence has been reached to collect the CM without this supplementation [103,120]. In order to prevent the contamination of the sample in question with serum proteins, in this type of approach, it is vital to do a proper washing of the cell culture [22].

The time that the MSCs are maintained in culture, and the number of passages they suffer before collecting the CM is another crucial variable. Controlling the time of culture is essential to prevent using cells that entered apoptosis [191] and, as a result, preventing a drop in the number of molecules of interest that they release and thus their therapeutic efficacy [192], as well as a rise in proteins that may have a detrimental impact on the tissues. Most authors use conditioning periods of 24 h to 72 h (Table 2), and the collection of the CM is followed by one or two centrifugations to eliminate cellular debris. A final consideration in the preparation of the CM is the amounts of secreted proteins present and the high dilution in the medium [21]. Thus, it is important to perform a concentration step that frequently involves ultrafiltration or molecular weight cut-off membranes ranging from 3 kDa to 100 kDa (Table 2), which also influences the final product obtained.

## 7. Current Limitations

As evidenced throughout the present review, the MSC secretome is composed of a variety of bioactive agents with beneficial effects in several models of brain diseases. However, there are still important challenges to the use of this tool in clinical practice.

The composition of the secretome is dependent on multiple variables, including the characteristics of the donor of the cells or MSCs’ tissue of origin [30]. Strikingly, the relationship between donor characteristics and secretome composition/therapeutic effects has been largely disregarded. On the other hand, concerning secretome production protocols, the variability is considerable. The different conditions used for cell culture (time in culture, cell density, number of cell passages, culture media, and supplements used), as well as the protocols for the preparation and concentration of the secretome, determine the composition of the product obtained, and, therefore, the final effects. Moreover, the fact that obtaining the final product of the secretome involves multiple steps, as well as the need to use supplements in the culture whose composition or origin is more difficult to control, increased the risk of contamination.

It is also important to note that although the CM may contain biomolecular material that leads to tissue regeneration, it remains a theoretical risk of immunogenicity due to the transfer of MHC molecules via extracellular vesicles [193]. This risk of immunogenic response may increase with a higher dose of secretome or its products, as was the case observed by Venugopal, C., et al. (2017). The authors observed that the administration of a high dose of exosomes had detrimental effects on neurons, not being able to protect against excitotoxicity and cell death. Also, when they analyzed the effects of administering these vesicles from both low-passage and high-passage MSCs, they identified that their efficacy decreased as the number of passages increased [192]. These results emphasize the need for a standardization of protocol and characterization and analysis of the dose to be administered. The lack of standardization of preparation and characterization protocols is a major obstacle to the regulatory approval of secretome use by The International Council for Harmonization of Technical Requirements for Pharmaceuticals for Human Use (ICH). Finding a guide to standardize the secretome’s composition, demonstrating its quality, stability, safety, and efficacy, will be an important point for its clinical use. The use of strategies for exhaustive characterization of the secretome, combined with the identification of markers of therapeutic potential, could help to overcome some of the limitations mentioned before. However, this is still a defiant strategy given the diversity of biomolecules forming the secretome, encompassing proteins, lipids, and RNA, as well as structures such as extracellular vesicles. Additionally, the processing and analysis of very small amounts of secreted proteins and the study of their pharmacokinetics and stability are challenging aspects of secretome research [194].

In addition to the need to standardize the protocols for secretome production, there is also the challenge of the costs associated with producing the large volumes needed to induce therapeutic benefits. In this respect, it is crucial to develop strategies that improve the delivery of the secretome to the brain and increase its stability, thus reducing the amount needed to promote repair. With this aim, optimizing dosing, delivery routes, and implementing new delivery systems are important factors currently being researched.

Even though most studies show that administering the secretome derived from MSCs has positive effects, few identified the main factors driving the therapeutic response, whether they reach the appropriate location, and which is the best way to protect it from degradation [55]. Despite the fact that MSCs have been shown to be able to home to injured sites [195], it is still not possible to determine whether the secretome also has the ability to reach these sites. Which secretome components can cross the BBB and reach the site of injury in the appropriate amount, composition, and timing is still unknown. Although there has already been evidence that exosomes reach the brain within 6 h after intranasal delivery [196], this is not known for other secretome components.

Different biomaterials are being developed to allow a sustained and controllable delivery of MSC secretome, limit its degradation, and thus improve therapeutic efficiency. Although there are numerous studies focusing on the use of alternative secretome delivery strategies based on hydrogels, scaffolds, lipophilic systems, or nanoparticles, whether functionalized or not, few studies have focused on the use of these strategies to deliver secretome to the brain. The need to surpass the blood–brain barrier creates an additional difficulty, limiting the use of most of these strategies for brain repair.

The administration of isolated EVs overcomes some of the problems identified above. Due to the propensity of EVs to be taken up by target cells through surface ligand/receptor interactions, membrane integrin adhesion, or endocytosis, the secreted factors contained in Evs are thought to be more stable than secreted factors that are free in solution [55]. However, the method used to isolate the specific products from the medium should also be considered. Ultracentrifugation, which is currently considered the standard method for isolating these structures, is capable of handling relatively large volumes; however, its recovery rates are low, and the process is time-consuming [197]. Methods such as size exclusion may carry other particles of similar size, resulting in reduced purity, and antibody-based capture methods can result in non-specific interference adsorption [198], have a high cost, and show low yields [199]. Microfluidic separation-based techniques, while allowing simultaneous isolation and characterization of Evs, have several challenges that still need to be overcome [200]. In addition, Yang, Y. et al. (2015), demonstrated that the internalization of MSC-derived exosomes was associated with the acquisition of new tumor cell properties by altering cellular functionalities and providing the capability to re-organize the tumor microenvironment [201]. In addition to the technical difficulties involved in isolating and purifying EVs, which have yet to be overcome, the use of isolated EVs eliminates components of the secretome that can play an important role in therapeutic effects.

**Figure 3:**
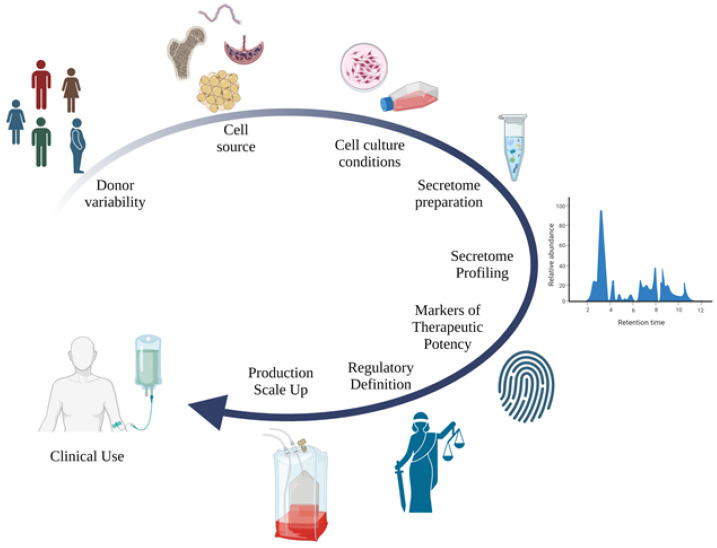
Steps required to establish conditions for the clinical use of secretome.Clarification of the impact of the cell donor and tissue of cell source, establishment of protocols for cell culture and secretome preparation, comprehensive characterization of the secretome, identification of markers of therapeutic potency, definition of regulations for the clinical application, and production scale up are all needed.

Finally, after the safe translation of these secretome-based therapies to clinical trials, it will be necessary to design protocols for large-scale production of secretome to support its therapeutic use without loss of potency and safety (Figure 3).

## 8. Conclusions

The secretome has been researched as an alternative to MSCs over the years. Its composition, characterized by several bioactive compounds that have neuroprotective, regenerative, and anti-inflammatory effects on the target tissue, has demonstrated effectiveness when applied to preclinical models of conditions that impact the central nervous system. Likewise, its preconditioning has demonstrated to have considerable potential by supporting the idea that when a stimulus is provided to the cells, they would respond by secreting important components, therefore promoting a higher potency.

Even though the use of the secretome still has numerous limitations, as is the case of the low standardization of protocols for its preparation, and although none of them are insuperable, additional research will be required to improve the chances of its clinical translatability.

## Figures and Tables

**Table 1:** Summary of the impact of different MSC preconditioning methods on the results obtained in different disease models.

Source	Model	Priming	Outcomes	Ref.
hP-MSCs	Stroke	3D culture/scaffolds	Functional recovery; ↓lesion volume; ↓cell death; ↑anti-inflammatory cytokines/neurotrophic factors; ↑neuronal differentiation; ↑angiogenesis;	[146]
hUC-CM	SCI	3D culture/scaffolds	Functional recovery; ↑angiogenesis; ↑remyelination	[137]
hUC-CM	TBI	3D culture/scaffolds	Functional recovery; ↓lesion volume; ↓cell death; ↓inflammation; ↓glial reactivity; ↑angiogenesis; ↑neurogenesis; ↑remyelination	[118]
hBM-MSCs-Evs	AD	3D culture/scaffolds	↓Glial reactivity	[136]
hBM-MSC-Ex	TBI	3D culture/scaffolds	Functional recovery; ↓inflammation; ↑angiogenesis; ↑neurogenesis	[49]
rBM-MSC	Stroke	Valproate and/or lithium chloride	Functional recovery; ↓lesion volume; ↑angiogenesis; ↑neurogenesis; ↑homing	[139]
hADSCs	MS	Rapamycin	↑Anti-inflammatory cytokines	[154]
hWJ-MSC	HIE	Thrombin	Functional recovery; ↓lesion volume; ↓cell death; ↓glial reactivity	[151]
hWJ-MSC	HIE	Thrombin	NA	[152]
rBM-MSCs	HI	Tetramethylpyrazine	Functional recovery; ↑angiogenesis; ↑homing	[149]
rBM-MSCs	Huntington	Valproate and/or lithium chloride	Functional recovery; ↓cell death	[147]
rBM-MSCs	PD	Fasudil	Functional recovery; ↑neurotrophic factors; ↑angiogenesis;	[153]
rBM-MSCs	Stroke	Hydrogen sulfide	Functional recovery; ↓lesion volume; ↓cell death; ↑neurotrophic factors	[148]
rBM-MSCs	Stroke	Roxadustat (FG-4592)	Functional recovery; ↓cell death; ↓inflammation; ↓glial reactivity;	[155]
rBM-MSCs	TBI	Calpain inhibitor (MDL28170)	Functional recovery; ↓lesion volume; ↓inflammation; ↓glial reactivity; ↑anti-inflammatory cytokines	[150]
rBMSC-CM	HIE	Cobalt chloride	Functional recovery; ↓cell death	[138]
rBMSC-CM	Neuroinflammation (induced by LPS)	Salidroside	↓Cell death; ↓inflammation; ↓glial reactivity	[156]
rBM-MSC	Stroke	IGF-1	Functional recovery; ↑angiogenesis;↑neurogenesis	[140]
rBM-MSCs	MS	SDF-1α	Functional recovery; ↓glial reactivity; ↑homing	[158]
hASC-CM	PA	TNF-α and IFN-γ	Functional recovery; ↓cell death; ↓inflammation; ↓glial reactivity; ↓oxidative Stress;	[162]
hASC-CM	TBI	TNF-α and IFN-γ	↓Inflammation; ↓glial reactivity; ↓loss of visual acuity	[160]
hASC-CM	TBI	TNF-α and IFN-γ	↓Inflammation; ↓glial reactivity; ↓loss of visual acuity;	[165]
hASC-CM	TBI	TNF-α and IFN-γ	↓inflammation; ↓excitoxicity	[159]
rASC-CM or hASC-CM	ICH	BDNF	↓Lesion volume; ↓glial reactivity	[164]
rBMSC-CM	Stroke	IL-1α	Functional recovery; ↓lesion volume; ↑body weight	[142]
hBM-MSC-Evs	AD	TNF-α and IFN-γ	↓Inflammation; ↓glial reactivity; ↑anti-inflammatory cytokines	[141]
rBM-MSC-Ex	TBI	BDNF	Functional recovery; ↓cell death; ↑neurogenesis	[163]
hADSCs	SCI	Hypoxia	↓Cell death; ↑neurogenesis	[169]
hOM-MSCs	ICH	Hypoxia	↓Cell death; ↓inflammation; ↓glial reactivity	[168]
rADMSCs	TBI	Hypoxia	Functional recovery; ↓cell death; ↓inflammation; ↑anti-inflammatory cytokines/neurotrophic factors;	[174]
rBM-MSCs	Brain injury caused by cardiac arrest	Hypoxia	↓Inflammation; ↑homing	[172]
rBM-MSCs	ICH	Hypoxia	Functional recovery; ↓lesion volume; ↑neurotrophic Factors; ↑neurogenesis;	[170]
rBM-MSCs	Stroke	Hypoxia	Functional recovery; ↓inflammation; ↑neurotrophic factors; ↑angiogenesis; ↑neurogenesis; ↑differentiation	[144]
rBM-MSCs	Stroke	Hypoxia	Functional recovery; ↓lesion volume; ↓cell death; ↑homing	[173]
rBM-MSCs	Stroke	Hypoxia	Functional recovery; ↑neurotrophic factors; ↑angiogenesis; ↑neurogenesis;	[171]
hASC-CM	TBI	Hypoxia	Functional recovery; ↓inflammation; ↓glial reactivity; ↓cell death	[178]
hBMSC-CM	TBI	Hypoxia	Functional recovery; ↓lesion volume; ↓cell death	[119]
rBMSC-CM	Stroke	Hypoxia	Functional recovery; ↓cell death; ↑angiogenesis	[177]
rBM-MSC-Ex	AD	Hypoxia	Functional recovery; ↓inflammation; ↓glial reactivity; ↑anti-inflammatory cytokines/neurotrophic factors	[143]
hUC-MSCs-Ex	Stroke	Hypoxia	Functional recovery; ↑body weight	[180]
hUC-MSCs-Ex	TBI	Hypoxia	Functional recovery; ↓cell death; ↓inflammation; ↑angiogenesis; ↑neurogenesis	[179]
rBM-MSCs	SCI	Low-intensity pulsed ultrasound	Functional recovery; ↓lesion volume; ↓glial reactivity; ↑neurotrophic factors	[184]
rBM-MSCs	Stroke	Ischemic brain tissue extract	↓Lesion volume; ↓cell death; ↑anti-inflammatory cytokines/neurotrophic Factors;	[181]
hUC-CM	TBI	Traumatically injured brain tissue extract	Functional recovery; ↓cell death; ↑neurotrophic factors; ↑neurogenesis; ↑homing;	[25]
hUC-MSCs-Ex	Stroke	Cerebral infarct tissue extracts	Functional recovery; ↓lesion volume; ↓cell death;	[183]

hP-MSCs—Human Placenta Mesenchymal Stem Cells; hUC-MSC—Human Umbilical Cord Mesenchymal Stem Cells; hUC-CM—Human-umbilical-cord-conditioned Medium; hUC-MSC-EX—Exosomes derived from Human Umbilical Cord; rBM-MSC—Rat Bone Marrow Mesenchymal Stem cells; rBM-CM—Rat-bone-marrow-conditioned Medium; hBM-MSC-Ex—Exosomes derived from Human Bone Marrow Mesenchymal Stem Cells; hBM-MSC-Ev—extracellular vesicles derived from Bone Marrow Mesenchymal Stem Cells; hOM-MSCs—Human Olfactory Mucosa Mesenchymal Stem cells; hASC—Human Adipose Tissue Mesenchymal Stem Cells; hWJMSC—Human Wharton’s Jelly Mesenchymal Stem Cells; NA—Not applicable; HIE—Hypoxic-ischaemic Encephalopathy; TBI—traumatic brain injury; SCI—spinal cord injury; MS—multiple sclerosis; AD—Alzheimer’s disease; ICH—Intracerebral Hemorrhage; PA—Perinatal Asphyxia; IGF-1—insulin-like growth factor 1; SDF-1α—Stromal Cell-derived Factor 1; TNF-α—tumor necrosis factor alfa; IFN-γ—Interferon gamma; BDNF—brain-derived neurotrophic factor; IL-1α—Interleukin 1-alpha; ↓—decrease; ↑cincrease.

**Table 2:** Summary of the different secretome preparation protocols, volumes administered, and number of administrations for the treatment of different neurological disease models.

Source	Model	Priming	Medium	Density	Conditioning Time	Concentration	T Volume Administered	N° Administrations	Ref.
rBMSC	AD	No	Serum-free RPMI	1 × 10^6^ cells	24 h	Ultrafiltration using centrifugal filters (unspecified)	25 μL	4 or 8	[88]
hBMSC	AD	No	Neurobasal-A	2.4 × 10^4^ cells/mL	24 h	INA	4 μL or 8 μL	1 or 2	[39]
SHED, BMSC and FibroMSC	AD	No	Serum-free DMEM	1 × 10^4^ cells/cm^2^	48 h	INA	400 μL	8	[89]
hWJMSC (differentiate into Ols)	EAE	No	Serum-free DMEM/F12	INA	72 h	100-fold with ultrafiltration using 3 kDa cut-off	140 μL	14	[96]
rASC	HIE	No	BME	4 × 10^6^ cells/cm^2^	24 h	250-fold by 10,000 cut-off	10 μL	1	[36]
PSC-EMSC	HIE	No	Serum-free α-MEM	9.2 × 10^4^ cells/cm^2^	24 h	Removal of ions and molecules below 1kDa	84 μL	7	[112]
hDPSC/hBMSC	Hippocampal neurodegeneration	No	Serum-free basal	NA	24 h	Not concentrated or diluted	8 μL	1	[90]
hBMSC	Impaired astrocytic exocytosis (transgenic model)	No	Neurobasal A	4.0 × 10^3^ cells/cm^2^	24 h	INA	0.5 μL	2	[131]
hUCMSC	NA	No	Serum-free DMEM	1 × 10^4^ cells/cm^2^	72 h	10-fold by 3 kDa cut-off	200 µL	1	[21]
hUCPVC	NA	No	Neurobasal A	4.0 × 10^3^ cells/cm^2^	24 h	INA	0.5 μL	1	[16]
rBMSC	Neuropathic pain	No	Serum-free DMEM	7 × 10^6^ cells	24 h	15-fold using ultrafiltration units (unspecified)	100 μL	1	[133]
rBMSC	SCI	No	DMEM with low glucose and with/FBS	5 × 10^3^ cells/cm^2^	24 h	INA	480 µL	16	[132]
hASC	Stroke	No	Serum-free α-MEM	INA	48 h	INA	0.5 μL	1	[103]
rBMSC	Stroke	No	DMEM/F12 supplemented with 2% LE rat serum	INA	24 h	10-fold by 5 kDa cut-off	INA	1	[102]
hESCMSC	Stroke	No	DMEM containing 0.05% human serum albumin and 2 mM L-glutamine, without FBS	INA	24 h	100-fold with 3 kDa cut-off	5 μL or 10 μL	1 or 2	[104]
hSHEDMSC, hBMSC	Stroke	No	Serum-free DMEM	4 × 10^5^ cells/cm^2^	48 h	INA	100 μL	13	[101]
hUCMSC	Stroke	No	Serum-free DMEM/F12	1 × 10^4^ cells/cm^2^	24 h	INA	140 μL	14	[100]
hBMSC	TBI	No	Serum-free DMEM	2 × 10^6^ cells/cm^2^	24 h	25-fold by 3 kDa cut-off	≈275 μL	INA	[37]
hBMSC	TBI	No	Serum-free DMEM	2 × 10^6^ cells/cm^2^	24 h	25-fold by 3 kDa cut-off	≈1699 μL	6	[119]
hUCMSC	TBI	No	Serum-free, low-glucose DMEM	2 × 10^6^cells	24 h	100 kDa cut-off (unspecified)	3 μL	1	[25]
hUCPVC	TBI	No	Serum-free EBM-2	2.5 × 10^3^ cells/cm^2^	72 h	NA	4 μL	1	[120]

hSHED—Human exfoliated deciduous teeth; hUCMSC—Human Umbilical Cord; hBMSC—Human Bone Marrow; hUCPVC—Human Umbilical Cord Perivascular Cells; hESCMSC—Human Embryonic stem cell-derived mesenchymal stem cells; hASC—Human Adipose Tissue; hPSC-EMSC—Human pluripotent stem cell-derived ectomesenchymal stem cells; hDPSCs—Human Dental Pulp; hWJMSC—Human Wharton’s Jelly; INA—Information not available; NA—Not applicable; DMEM—Dulbecco’s Modified Eagle Medium; EBM—Endothelial Basal Medium; RPMI—Roswell Park Memorial Institute Medium; BME—Basal Medium Eagle; HIE—Hypoxic-ischaemic Encephalopathy; TBI—traumatic brain injury; SCI—spinal cord injury; EAE—Experimental Autoimmune Encephalomyelitis; AD—Alzheimer’s disease.

## Data Availability

Not applicable.

## Abbreviations

α-MEM	alpha minimal essential medium
AD	Alzheimer’s disease
ADSCs	adipose tissue
BBB	Blood–brain barrier
BDNF	brain-derived neurotrophic factor
bFGF	basic fibroblast growth factor
BME	Eagle’s basal medium
BM	bone marrow
CM	conditioned medium
CNS	central nervous system
DMEM	Dulbecco’s Modified Eagle’s Basal Medium
EVs	extracellular vesicles
Gal-1	galectin-1
GDNF	glia-derived growth factor
HIE	Hypoxic-ischemic Encephalopathy
HGF-1	hepatocyte growth factor 1
HSP	heat shock protein
IFN-Y	interferon
IGF-1	insulin-like growth factor type 1
IL	interleukin
IL-1Ra	interleukin 1 receptor antagonist
miRNA	Micro-ribonucleic acid
mRNA	Messenger ribonucleic acid
MSCs	mesenchymal stem cells
MVs	microvesicles
NGF	nerve growth factor
TBI	traumatic brain injury
TGF-B	tumor growth factor B
TNF-a	tumor necrosis factor α
TSG101	tumor susceptibility protein 101
VEGF	vascular endothelial growth factor
